# HBV Infection Induces Changes in Mitochondrial Architecture and Remodeling of Mitochondria–ER Interactions

**DOI:** 10.3390/pathogens15101009

**Published:** 2026-09-24

**Authors:** Leonardo Duca, Carlotta Castelli, Anna Rosa Garbuglia, Laura Falasca

**Affiliations:** 1 Laboratory of Electron Microscopy, National Institute for Infectious Diseases Lazzaro Spallanzani—IRCCS, 00149 Rome, Italy; leonardo.duca@inmi.it (L.D.); carlotta.castelli@inmi.it (C.C.); 2 Laboratory of Virology, National Institute for Infectious Diseases Lazzaro Spallanzani—IRCCS, 00149 Rome, Italy; annarosa.garbuglia@inmi.it

**Keywords:** HBV, mitochondria, electron microscopy, confocal microscopy, endoplasmic reticulum, mitochondria–ER contacts, MERCs, HepG2-NTCP cells

## Abstract

Mitochondria are central hubs of cellular metabolism, playing essential roles in energy production, lipid metabolism, calcium homeostasis, and redox signaling. Findings show that many of these activities are regulated by contact sites between mitochondria and the endoplasmic reticulum (ER) (mitochondria–ER contacts, MERCs). Several studies suggest that Hepatitis B Virus (HBV) can affect mitochondrial functions, inducing metabolic alterations and oxidative stress. This work aimed to investigate, at an ultrastructural level, whether HBV infection causes changes in mitochondrial architecture and MERC modulation. Using HepG2-NTCP cells as an in vitro model system, we analyzed mitochondrial modifications after HBV infection at 2, 5, and 10 days. Electron and confocal microscopy analyses highlighted changes in mitochondrial morphology with a progressive enlargement of mitochondrial size and a remodeling of the mitochondrial network, together with an increase in MERC establishment. Moreover, functional assays showed decreased ATP production, despite increased mitochondrial membrane potential and tight regulation of mitochondrial reactive oxygen species (mtROS) levels. These findings suggest that HBV does not cause irreversible mitochondrial damage but drives a dynamic and coordinated adaptation involving metabolism, ultrastructure, ER interaction, and network organization.

## 1. Introduction

Hepatitis B virus (HBV) infection represents one of the major global health problems, with approximately 300 million people chronically infected and nearly one million deaths annually due to complications directly linked to the infection. There is currently no definitive cure for the chronic form of the disease, which can only be prevented through vaccination [1]. The chronic form of the disease can lead to severe complications, including cirrhosis and hepatocellular carcinoma (HCC).

HBV is a small DNA virus belonging to the Hepadnaviridae family that specifically targets hepatocytes. Upon entering the liver cell, the viral DNA is released and transported into the host cell nucleus. Once inside, the viral DNA is transformed into covalently closed circular DNA (cccDNA), which serves as a template for viral replication. Viral cccDNA is stable and extremely difficult to eradicate; it persists in the nucleus, where it can also integrate into the host DNA [2,3]. Because of its small DNA dimension, HBV has limited coding capacity; consequently, it must hijack host cellular components to sustain its life cycle and evade intracellular defenses. HBV infection forces changes in key hepatocyte metabolic pathways and exploits glucose/lipid metabolism [4,5]. Systemic metabolic dysregulation in patients infected with HBV correlates with disease progression and HCC risk [6,7]. Serum metabolomic profiling of chronic hepatitis B (CHB) patients reveals a distinct viral-induced signature characterized by significantly elevated circulating levels of lactate, pyruvate, and free fatty acids, which progressively increase as patients advance to chronic active hepatitis phases [8]. Intrahepatic and experimental analyses demonstrate that HBV can promote intracellular cholesterol accumulation in hepatocytes and substantially remodel hepatic lipid metabolism [9]. Hepatocyte metabolic physiological functions are highly dependent on mitochondria. Mitochondria serve as the central hub for the tricarboxylic acid (TCA) cycle and oxidative phosphorylation (OXPHOS), generating the ATP supply required for vital hepatic processes such as gluconeogenesis, urea synthesis, and plasma protein secretion [10]. Beyond energy production, hepatic mitochondria coordinate systemic metabolic homeostasis by executing fatty acid β-oxidation during fasting states. This intense metabolic activity is tightly coupled with a robust antioxidant defense system to neutralize the high levels of physiological reactive oxygen species (ROS) produced by the hepatic respiratory chain, ensuring cellular survival and systemic metabolic stability [11].

Several studies suggest that HBV can affect mitochondrial functionality through the HBx protein. HBx is able to interact with the outer mitochondrial membrane, inducing oxidative stress and changes in mitochondrial metabolism that ultimately contribute to cellular dysfunction and aberrant mitochondrial dynamics [12]. Mitochondrial dynamics represent changes in mitochondrial architecture to adapt to variations in bioenergetic demands, regulating cellular metabolic homeostasis [13]. Key regulators of mitochondrial functions are highly dynamic inter-organelle junctions where the outer mitochondrial membrane and the ER are held in close apposition, named Mitochondria–ER Contact Sites (MERCs) [14,15].

The aim of this work was to characterize the impact of HBV infection on mitochondrial architecture and mitochondria–ER interactions. A cell culture model system was utilized, which enables infection with complete HBV. This experimental model allows us to deepen current knowledge of the impact of HBV on mitochondria, which mostly comes from studies based on HBx overexpression rather than from true in vitro infections [16]. Mitochondrial function, ultrastructure, and organelle organization were simultaneously analyzed throughout the temporal course of HBV infection.

## 2. Materials and Methods

### 2.1. Cell Culture

HepAD38 cells (CRL-3561, ATCC, American Type Culture Collection, Manassas, VA, USA), a stably transfected hepatoblastoma cell line that replicates human HBV (genotype D, subtype ayw) under the control of a tetracycline-regulated promoter (Tet-off) [17] were maintained in Dulbecco’s Modified Eagle’s Medium Nutrient Mixture F-12 (DMEM F-12, Cat. No. D6421, Sigma-Aldrich, St. Louis, MO, USA) supplemented with 10% Fetal Bovine Serum (FBS, Cat. No. A5670701, Gibco, Carlsbad, CA, USA), with GlutaMAX^TM^ (Cat. No. 35050061, Gibco, Carlsbad, CA, USA), Penicillin G/Streptomycin (Cat. No. SIAL- PEN/STREP, S.I.A.L. Srl, Rome, Italy), 400 μg/mL of G418 sulfate (Cat No. 30-234-CR, Corning, Corning, NY, USA), 50µM of hydrocortisone–hemisuccinate (Cat. No. H2270, Sigma-Aldrich, St. Louis, MO, USA), and 0.3 µg/mL of tetracycline 0.3 µg/mL (Cat. No. T7660, Sigma-Aldrich, St. Louis, MO, USA) at 37 °C and 5% CO_2_. To produce infectious HBV particles, HepAD38 were cultured in medium without tetracycline.

HepG2-NTCP cells (Cat. No. T6190, Applied Biological Materials Inc., Richmond, BC, Canada), derived from HepG2 cells to overexpress human Sodium Taurocholate Co-Transporting Polypeptide (NTCP), were used for HBV infection [18]. HepG2-NTCP cells were grown in Dulbecco’s Modified Eagle’s Medium (DMEM, Cat No. 15353551, Corning, Corning, NY, USA) supplemented with 10% FBS (Cat. No. A5670701, Gibco, Carlsbad, CA, USA), GlutaMAX^TM^ (Cat. No. 35050061, Gibco, Carlsbad, CA, USA), Penicillin G/Streptomycin (Cat. No. 35050061, Gibco, Carlsbad, CA, USA), 300 µg/mL of G418 sulfate (Cat No. 30-234-CR, Corning, Corning, NY, USA), and they were incubated at 37 °C and 5% CO_2_.

### 2.2. HBV Infection

HepG2-NTCP cells were infected with a multiplicity of infection (MOI) of 500 VGE/cell (viral genome equivalent/cell) in DMEM (Cat No. 15353551, Corning, Corning, NY, USA) with 3% FBS (Cat. No. A5670701, Gibco, Carlsbad, CA, USA), 300 µg/mL of G418 sulfate (Cat No. 30-234-CR, Corning, Corning, NY, USA), 1% dimethyl sulfoxide (DMSO, Cat No. 15303671, Corning, Corning, NY, USA), 4% polyethylene glycol 8000 (PEG 8000, Cat. No. 89510-250G-F, Sigma-Aldrich, St. Louis, MO, USA) at 37 °C and 5% CO_2_. After 24 h of adsorption, viral inoculum was removed, and fresh medium containing 3% FBS was added. Cells were maintained in culture up to 10 days post-infection (dpi), and the medium was replaced with fresh medium every two days. Uninfected (UN) control cells were cultured in parallel and maintained in the same medium containing 1% DMSO throughout the experiment.

### 2.3. HBV-DNA Extraction and Quantification

For extracellular HBV-DNA quantification, supernatants were collected at different time points (2, 3, 5, 7, and 10 dpi) and analyzed using the Aptima® HBV Quant Assay (Hologic Inc., Marlborough, MA, USA) on the fully automated Panther® System. For intracellular HBV-DNA level quantification, cells were collected at different time points (2, 5, and 10 dpi), and DNA was extracted using Quick-DNA^TM^ MicroPrep (Cat. No. D3020, Zymo Research, Irvine, CA, USA) and quantified using the Aptima® HBV Quant Assay.

### 2.4. Transmission Electron Microscopy Analysis

Transmission electron microscopy (TEM) was performed on cultured cells using standard procedures. Uninfected and infected HepG2-NTCP cells (at 2, 5, and 10 dpi) were fixed with 2.5% glutaraldehyde (Cat. No. 01909, Polysciences, Warrington, PA, USA) in 0.1 M cacodylate (Cat. No. C4945, Sigma-Aldrich, St. Louis, MO, USA) buffer at 4 °C. Samples were then post-fixed with 1% osmium tetroxide (OsO_4_) (Cat. No. 75632, Sigma-Aldrich, St. Louis, MO, USA), dehydrated in graded ethanol (Cat. No. 02860, Fluka, Seelze, Germany) and embedded in Epon resin (Cat. No. 45359-1EA-F, Sigma-Aldrich, St. Louis, MO, USA). Ultrathin sections were stained with 2% uranyl acetate (Cat. No. R1260A, Agar Scientific, Rotherham, UK) and observed under a JEOL JEM 2100 Plus Transmission Electron Microscope (Japan Electron Optics Laboratory Co., Ltd., Tokyo, Japan). Images were digitally captured with a digital camera TVIPS (Tietz Video and Image Processing Systems GmbH, Gauting, Germany).

### 2.5. Confocal Microscopy Analysis

Immunofluorescence and confocal microscopy analysis were performed for staining of viral proteins and for visualization of mitochondria. To this aim, cells were cultured in 2-well glass chamber slides (Cat. No. 177380, Nunc, Lab-Tek, Waltham, MA, USA). At different time points (2, 5, and 10 days), uninfected and infected HepG2-NTCP cells were fixed with 4% paraformaldehyde (Cat. No. P-6148, Sigma-Aldrich, St. Louis, MO, USA). Samples were washed in Phosphate-Buffered Saline (Cat. No. 18912014, PBS, Gibco, Carlsbad, CA, USA) and permeabilized for 1 h in PBS containing 0.1% Triton X-100 (Cat. No. 93418, Fluka, Seelze, Germany), 1% Bovine Serum Albumin (Cat. No. A7906, BSA, Sigma-Aldrich, St. Louis, MO, USA), and 10% Normal Goat Serum. Primary antibodies: rabbit anti-Tom20 (a mitochondrial-localized protein) antibody (Cat. No. 11820-1-AP, Proteintech, Rosemont, IL, USA), rabbit polyclonal antibody anti-hepatitis B virus core antigen (Cat. No. 216A-17-RUO, Cell Marque, Rocklin, CA, USA) were incubated overnight at 4 °C. After washing, samples were incubated with Alexa Fluor 488 and Alexa Fluor 647 conjugated secondary antibodies (Molecular Probes, Grand Island, NY, USA). Confocal fluorescence microscopy images were obtained using a ZEISS 900 LSM confocal microscope (ZEISS, Oberkochen, Germany) with an Airyscan2 detector and processed using ZEISS Zen 2 software (ZEISS, Oberkochen, Germany).

### 2.6. ATP Measurement

ATP levels were measured using the CellTiter-Glo^®^ Luminescent Cell Viability Assay kit (Cat. No. G9241, Promega Corporation, Madison, WI, USA). Cells (uninfected and infected cells at 2, 5, and 10 dpi) seeded in 96-well plates (Cat. No. 6505098, Greiner, Kremsmünster, Austria) at 80.000 cells/well were treated according to the manufacturer’s instructions. Luminescent signal was quantified using a Cytation 5 Cell Imaging Reader (BioTek Instruments, Winooski, VT, USA), and ATP levels were calculated according to the standard curve.

### 2.7. Mitochondrial Membrane Potential Analysis

Mitochondrial membrane potential was analyzed using the Mitochondrial Membrane Potential Kit (Cat. No. MAK159, Sigma-Aldrich, St. Louis, MO, USA) according to the manufacturer’s instructions. Cells were seeded in 96-well plates (Cat. No. 6505090, Greiner, Kremsmünster, Austria) at a density of 80.000 cells/well. Experimental conditions included uninfected and infected cells for 2, 5, and 10 dpi. Fluorescence was quantified using a Cytation 5 Cell Imaging Reader (BioTek Instruments, Winooski, VT, USA), and the Mitochondrial Membrane Potential was calculated as the ratio between red and green fluorescence and normalized to the control samples.

### 2.8. Mitochondrial ROS (mtROS) Measurements

mtROS production was determined using MITOSOX^TM^ Red Mitochondrial Superoxide Indicator (Cat. No. M36008, Invitrogen, Carlsbad, CA, USA) according to the manufacturer’s instructions. Cells were plated and treated as described for the mitochondrial membrane potential analysis. Fluorescence was quantified using a Cytation 5 Cell Imaging Reader (BioTek Instruments, Winooski, VT, USA), and values were normalized to the control samples.

### 2.9. TEM Images Morphometric Analysis

Mitochondria measurements on TEM micrographs were obtained using ImageJ/Fiji software (fiji-windows-x64, National Institutes of Health, Bethesda, MD, USA) by manually tracing outlines of mitochondria. These regions of interest (ROI) enabled the automated quantification of morphological parameters [19]. Measurements were performed on at least 40 mitochondria per independent replicate (*N* = 3) for each experimental condition. Different morphometric descriptors were analyzed for mitochondrial size (Area, Perimeter) and mitochondrial shape (Circularity, Roundness, Aspect Ratio (AR; major-to-minor axis)). The proportions of MERCs were measured by drawing the lines spanning the length of the ER-contact site and measuring the distance between the two organelles. In addition, the ER–mitochondrial Contact Coefficient (ERMICC) was calculated as the interface length divided by the product of the mitochondrial perimeter and the ER–mitochondria distance [20].

### 2.10. Confocal Microscopy and Fluorescence Imaging Morphometric Analysis

Confocal fluorescence images, for visualization and quantification of the mitochondrial network, were captured using a 63× oil objective. Three-dimensional (3D) confocal z-stacks were acquired and subsequently subjected to image deconvolution. These deconvolved 3D datasets were then processed using ImageJ/Fiji software (fiji-windows-x64, National Institutes of Health, Bethesda, MD, USA). Using the binary images (white mitochondria/black background), individual mitochondrial particles were analyzed with the software’s “Analyze Particles” function to calculate Circularity, Form Factor (FF), and AR. We calculated the degree of Mitochondrial Fragmentation Counts (MFCs) for each imaged cell by dividing the mitochondrial number by the total mitochondrial area and arbitrarily multiplying by 1000, following a previous report [21]. In addition, 3D movies were generated from the image stacks to successfully visualize spatial mitochondrial distribution.

### 2.11. Use of Generative Artificial Intelligence (GenAI)

The authors used generative artificial intelligence (GenAI) tools, including ChatGPT (GPT-5.5, OpenAI, San Francisco, CA, USA), to assist with graphical abstract preparation. The authors have reviewed and edited the output and take full responsibility for the content of this publication.

### 2.12. Statistical Analysis

We calculated *p*-values using Prism 9 (GraphPad Software, La Jolla, CA, USA) as specified in the figure legends; *p*-values of less than 0.05 were considered statistically significant; * *p* < 0.05, ** *p* < 0.01, *** *p* < 0.001, **** *p* < 0.0001. Statistical significance was determined based on independent biological replicates (*N* = 3), utilizing the average value per independent experiment for comparison. Statistical analyses were performed using the Kruskal–Wallis test for non-normally distributed data and the Brown–Forsythe and Welch ANOVA tests for normally distributed data, whereas categorical data were analyzed using the chi-square test. Lastly, Spearman’s correlation coefficient was used to assess the correlation between variables.

## 3. Results

### 3.1. HBV Infection of NTCP-Cells

To determine viral production titer, culture medium from HBV-infected HepG2-NTCP cells was collected at 2, 3, 5, 7, and 10 dpi, and HBV-DNA was quantified by qPCR. The results obtained show that viral particle release was higher during the first three days, whereas viral titers declined at 5 dpi and were drastically reduced at 10 dpi (Figure 1A).

However, we cannot rule out that residual virus remaining from the inoculum could contribute to the total amount of HBV-DNA signal at early time points. The intracellular kinetics of HBV were analyzed by qPCR of total HBV-DNA in cell lysates. A strong decline of intracellular HBV-DNA was observed from 2 to 5 dpi, followed by a rapid increase from 5 to 10 dpi (Figure 1B). Immunofluorescence staining for HBcAg confirmed cell infection, with an increase in the proportion of positive cells over time (Figure S1).

Transmission electron microscopy was used to inspect infected cells to visualize HBV-like particles. Although detection of HBV virions in the cytoplasm of cells was difficult, some spherical HBV-like particles (around 45–50 nm in diameter) were found in the perinuclear area, enclosed in cytoplasmic vesicles (Figure 2A).

Single particles were occasionally observed near the cell surface, and even though direct budding of the virus at the cell membrane was not observed, HBV-like particles were visualized in very small vesicles (likely exosomes) outside the cell (Figure 2B). Viral-like particles were also found inside multivesicular bodies (MVBs), which, together with lipid droplets, increased and appeared as a consequence of infection (Figure 2C,D).

### 3.2. HBV Remodeling of Mitochondrial Morphology

TEM analysis of uninfected control cells showed a cytoplasm rich in mitochondria (generally presenting an elliptical shape) and well-organized ER cisternae, as typical for hepatocytic cells [22] (Figure 3A). These features appeared unaffected by culture duration (Figure S2).

During HBV infection, we observed changes in both mitochondrial morphology and the extent of ER contacts over time (Figure 3B–D). For this reason, ultrastructural morphometric analysis was performed in single sections of cells, evaluating different parameters to describe mitochondrial morphology in detail.

The analysis of more than 120 mitochondria per condition (uninfected; infected at 2, 5, and 10 dpi) revealed that mitochondria in infected cells became progressively larger compared with the normal uninfected condition (Figure 4).

In particular, at 10 dpi, mitochondrial area and perimeter appeared approximately double those of mitochondria in uninfected cells (Figure 4A,B; Table 1).

Nevertheless, at this time point of HBV infection, the shape parameters (Circularity, AR, and Roundness) remained essentially unchanged compared with uninfected cells (Figure 4C–E), overall suggesting an isotropic mitochondrial hypertrophy. Mitochondria are not elongating (pure fusion), and they are not ballooning into perfect spheres (pure osmotic swelling); instead, they are growing uniformly in all directions. These results were confirmed by qualitative observations of TEM images (Figure 3D).

In contrast, morphometric analysis showed that mitochondria at 2 dpi exhibited a significant decrease in Circularity compared with mitochondria in uninfected cells (Figure 4E), which suggested that at early time post-infection mitochondria could become more elongated.

### 3.3. HBV-Induced Modification of Mitochondria–ER Interactions

Ultrastructural EM observation also highlighted modifications associated with HBV infection concerning the extent of ER–mitochondria contacts (Figure 3). Uninfected HepG2-NTCP cells displayed an abundance of contacts between rough ER and mitochondria (Figure 3A); however, the length of the interaction between the two organelles appeared affected by the presence of the virus. Quantification of the interface between ER and mitochondria revealed that in uninfected cells, 80.9% of mitochondria exhibited interactions with ER membranes (Table 2), with membrane contacts covering up to 63% of the mitochondrial perimeter (median of 29.6%) (Figure 5A).

HBV infection markedly increased the proportion of ER-associated mitochondria: up to 93.6% of mitochondria in cells after 2 dpi; 98.1% of mitochondria in cells at 5 dpi; and 100% at 10 dpi, indicating association with rough ER (Table 2). Moreover, striking differences concern the length of ER in contact with the mitochondrial perimeter, particularly pronounced at 10 dpi (median mitochondrial coverage, 71,4%) (Figure 5A). Of note, at 2 dpi and 10 dpi, we observed mitochondria fully enveloped by ER membranes (Figure 5D,E) (Table 3).

Swollen ER membrane was occasionally observed at 10 dpi (Figure 3D). No significant differences were found in the distance between the ER membrane and the mitochondrial surface between uninfected and infected cells (Figure 5B); nevertheless, the ERMICC was found to significantly increase in HBV-infected cells as compared with uninfected cells (two-fold increase at 2 and 10 dpi) (Figure 5C).

### 3.4. Mitochondrial Network Imaging and Morphometric Analysis

Since electron microscopy investigations showed evidence of mitochondria–ER modifications, we aimed to clarify whether this might be reflected in changes in the spatial mitochondrial network. To ensure precise visualization of mitochondrial spatial distribution, fluorescent labeling of mitochondria was performed on uninfected and infected cells at different times after HBV infection; super-resolution confocal Airyscan microscopy was used, and acquired z-stacks were subsequently processed for image morphometric quantification and 3D reconstruction.

Mitochondria in uninfected cells were mostly distributed in the perinuclear area and formed a well-connected network (Figure 6A, Video S1).

Visual analysis of these morphological features highlighted changes in mitochondrial shape and 3D interconnection induced by HBV infection (Figure 6B–D, Videos S2–S4).

To ensure precise quantification of the mitochondrial network, ImageJ/Fiji software was used, and different parameters were measured. The AR was significantly higher in cells at 2 dpi (*p* < 0.001 and *p* < 0.01 vs. 5 and 10 dpi, respectively) (Figure 7A).

At 10 dpi, we found a strong reduction in the FF (10 dpi vs. 2 dpi, *p* < 0.001) (Figure 7B) and a significant increase in the MFC (Figure 7C), indicating that mitochondrial structure was less elongated and more fragmented (i.e., less interconnected).

### 3.5. Mitochondrial Functional Remodeling Induced by HBV

To evaluate whether mitochondrial morphological changes induced by HBV were associated with mitochondrial functional impairment, ATP content, ΔΨm and mtROS were investigated. Quantification of intracellular ATP levels revealed that ATP production progressively declined during infection. Compared with uninfected cells, a significant decrease in ATP levels was already detectable at 2 dpi, becoming even more pronounced at later time points, particularly at 5 dpi, suggesting that HBV infection could affect bioenergetic homeostasis (Figure 8A).

Modification of ΔΨm was assessed using the JC-10 fluorescent probe. Quantification of the fluorescence ratio demonstrated a progressive increase in ΔΨm during infection. Significant variation was detected at 2 dpi compared with uninfected cells, and a further increase was found at both 5 and 10 dpi (Figure 8B), indicating that HBV profoundly affects mitochondrial polarization dynamics despite a decline in ATP production. We next evaluated mitochondrial ROS production. MitoSOX staining quantification showed that while ROS production was reduced at 2 dpi, a significant increase was observed at 5 dpi, before returning close to the levels of uninfected cells at 10 dpi (Figure 8C). These findings suggested that mitochondrial oxidative stress is tightly regulated during HBV infection rather than progressively accumulating.

### 3.6. Correlation Between Mitochondrial Modifications and Days of Infection

Correlation analysis was performed on parameters that had shown higher statistical significance. The results revealed a progressive association between infection time and changes in both mitochondrial morphology and function (Table 4).

Among the morphometric parameters, the percentage of apposition showed the strongest positive correlation with infection time. Significant positive correlations were also observed for ERMICC and mitochondrial fragmentation count, while mitochondrial area and perimeter displayed more moderate associations. Notably, mitochondrial functional parameters showed a strong correlation with infection time. ATP levels were strongly inversely correlated with infection time, whereas ΔΨm showed a strong positive correlation, and a moderate positive correlation was observed for mtROS levels. Overall, these findings indicate that longer infection times are associated with coordinated alterations in mitochondrial morphometric and functional parameters.

## 4. Discussion

HBV has evolved multiple strategies to exploit host–cell metabolism and organelle dynamics in order to establish a productive infection [5]. Mitochondria represent one of the principal intracellular targets of HBV; in particular, the HBx protein, which localizes to the outer mitochondrial membrane, has been implicated in regulating mitochondrial bioenergetics, calcium signaling, mtROS production, and mitophagy [12,23,24]. However, several research gaps remain because in vitro overexpression models force HBx to accumulate in the cytoplasm and mitochondria; conversely, in natural infection, HBx is expressed at very low levels and localizes predominantly to the nucleus to regulate the cccDNA minichromosome [25].

The present study strongly suggests that HBV infection profoundly remodels mitochondrial biology in HepG2-NTCP cells through coordinated alterations affecting mitochondrial function, morphology, and ER–mitochondria communication.

Our findings suggest that HBV induces an early metabolic adaptation. ATP production decreases already during the initial stages of infection, and although complete bioenergetic failure was not detected, infected cells at later stages of infection displayed a persistence of lower ATP availability. One would expect a concomitant loss of membrane potential and increased oxidative stress; instead, mitochondria in infected cells even increased mitochondrial polarization. Such bioenergetic uncoupling (increase in ΔΨm and simultaneous decrease in ATP concentration) has been reported in several chronic viral infections and may represent a strategy by which HBV allows infected cells to preserve mitochondrial integrity despite altered metabolic demands [26,27]. This interpretation appears consistent with mtROS production. During the earliest phase of infection, we found that mtROS tends to decrease, whereas a transient increase becomes detectable at intermediate time points, before returning toward basal values. This observation agrees with increasing evidence indicating that moderate mtROS production acts as a signaling mechanism during HBV infection rather than representing simply a cytotoxic by-product. Controlled ROS levels may contribute to activation of signaling pathways involved in viral persistence while avoiding excessive oxidative damage [28]. This suggests that HBV imposes a significant metabolic burden on the host cell while preserving sufficient energetic capacity to sustain both cellular survival and viral persistence.

Ultrastructural analyses revealed substantial mitochondrial remodeling. Both mitochondrial area and perimeter progressively increase during infection. This mitochondrial enlargement is unlikely to reflect osmotic swelling; instead, it appears compatible with active remodeling of mitochondrial architecture. The induction of larger mitochondria is usually driven primarily by increased cell energy demands or environmental stress [29]. Some viral infections, such as influenza or Dengue, can induce mitochondrial enlargement and active inhibition of mitochondrial fission to optimize OXPHOS to meet viral energy demands [30]. Thus, the enlargement of mitochondrial size we observed could be not merely a consequence of HBV infection but rather a virus-driven strategy to optimize the intracellular environment for efficient replication. Indeed, HBV replication has been demonstrated to be closely associated with extensive metabolic reprogramming in infected cells, due to energetic and biosynthetic demands of viral propagation [31]. Metabolomic analyses show that HBV infection increases glucose uptake and promotes a metabolic shift toward aerobic glycolysis, resembling the Warburg effect, while simultaneously enhancing flux through the tricarboxylic acid cycle and the pentose phosphate pathway. Beyond glucose metabolism, HBV also remodels lipid, amino acid, and mitochondrial metabolism, establishing a coordinated metabolic network that supports viral protein synthesis, virion production, and viral persistence [5].

One of the most interesting findings of this study concerns the marked increase in ER–mitochondria interactions. MERCs play essential roles in cell metabolism and inflammatory signaling [32]. Therefore, expansion of MERCs may represent a central mechanism by which HBV reshapes host–cell functions. Of note, our ultrastructural and morphometric analysis highlighted an increased proportion of mitochondria surrounded by ER membranes along their entire perimeter. This kind of association resembles the wrapper-ER described by Anastasia et al. in liver hepatocytes [33], which envelops mitochondria to manage lipid homeostasis. In this context, HBV and lipid metabolism are closely linked, since HBV relies on host cell cholesterol and lipoproteins to assemble its viral particles [34]. However, a limitation of this work is that our two-dimensional ultrastructural analysis does not allow us to determine the extent and organization of these contacts or establish whether they correspond to continuous sheets of ER wrapping around the mitochondrial surface, as demonstrated by 3D electron tomography in [33].

Normally, mitochondria form a well-connected network that can be described as large and branched clusters. Research findings have demonstrated that fragmentation of the mitochondrial network is implicated in metabolic diseases, like type 2 diabetes and obesity [35,36]. Confocal microscopy, in addition to supporting the ultrastructural observations, provides evidence of increased mitochondrial network fragmentation, further indicating that HBV infection progressively reorganizes the mitochondrial network complexity.

We cannot completely exclude a potential contribution of other HepAD38-derived components, such as proteins or metabolites, present in the viral inoculum during the early stages of infection; nevertheless, our findings indicate that all changes become particularly evident during the later stages of infection. This temporal sequence suggests that mitochondrial network remodeling is not simply a consequence of active viral replication. Instead, it may suggest that mitochondrial remodeling is not solely linked to active viral production but persists as a consequence of cellular reprogramming triggered by the infection. The maintenance of altered mitochondrial architecture despite reduced viral burden indicates that HBV induces durable modifications of organelle homeostasis.

## 5. Conclusions

Overall, the findings support a model in which HBV infection does not simply induce mitochondrial damage; rather, it promotes a progressive mitochondrial reprogramming that may contribute to viral persistence and long-term cellular dysfunction. Future investigations will be performed to evaluate the impact of different HBV genotypes, trying to explain why certain viral genotypes cause more aggressive liver pathogenesis than others.

## Figures and Tables

**Figure 1:**
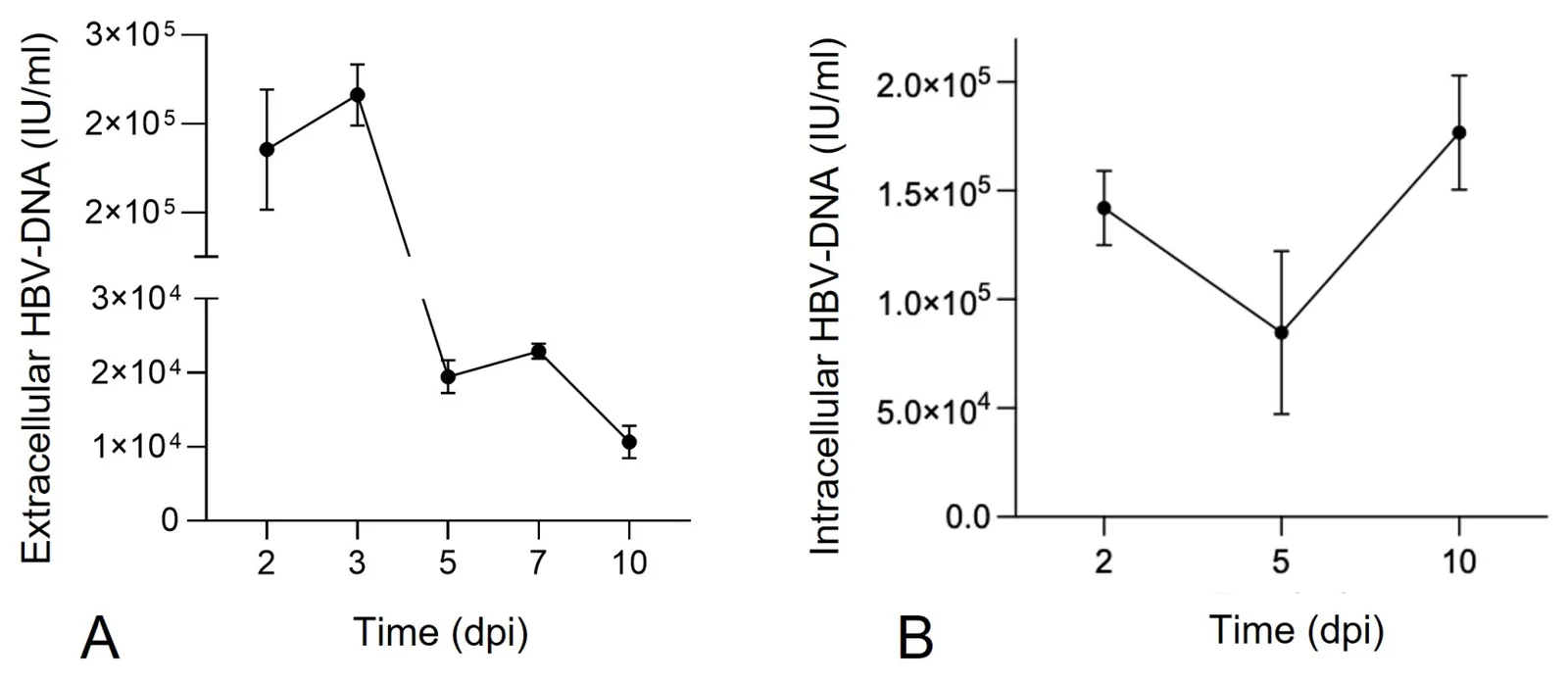
Quantification of (**A**) extracellular and (**B**) intracellular HBV-DNA levels by qPCR in HepG2 NTCP cells at different days post infection (dpi). Each time point includes at least three biological replicates. Data are presented as mean values ± standard deviation (SD).

**Figure 2:**
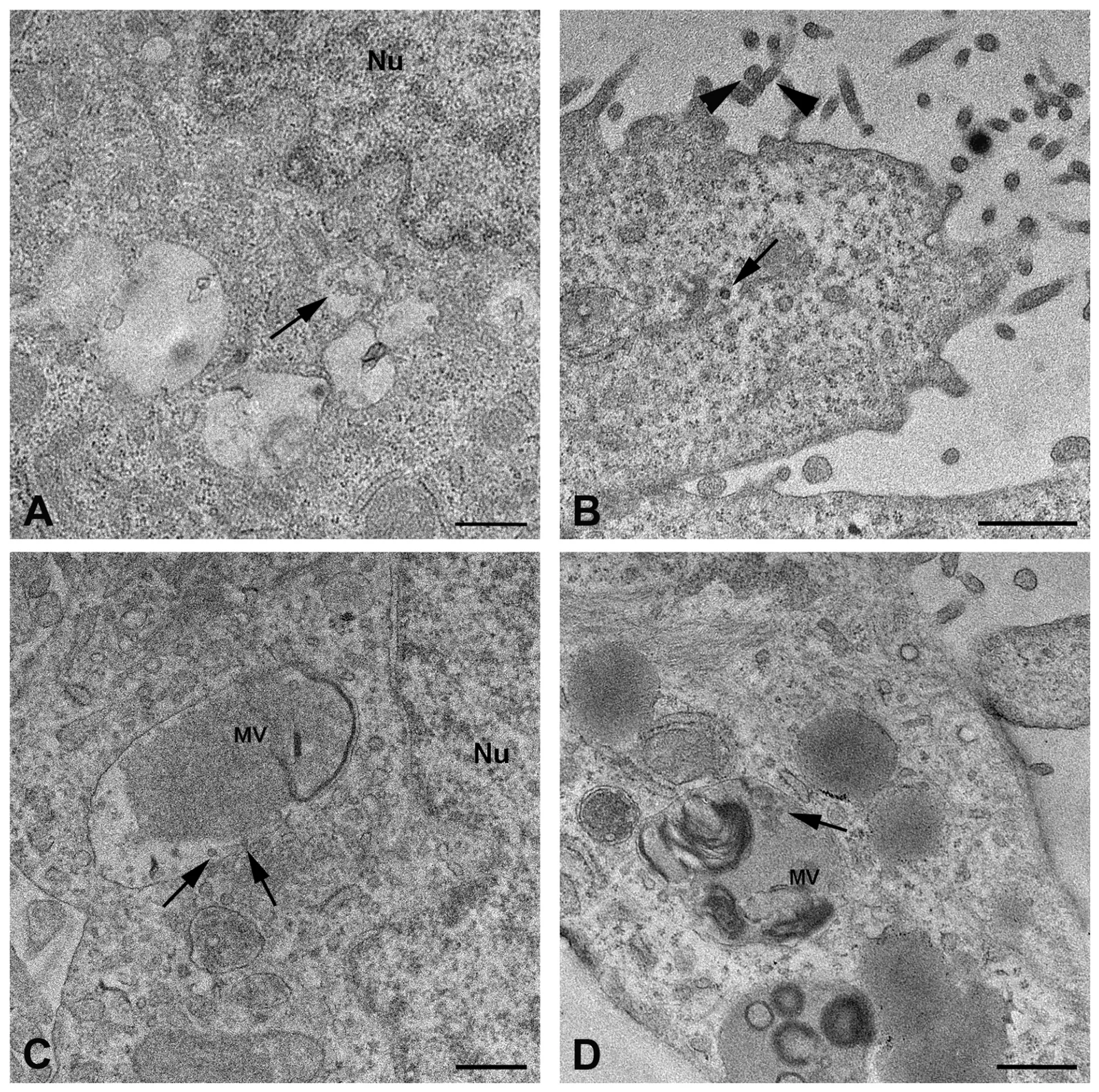
Transmission electron micrographs showing HBV-like particles in infected HepG2 NTCP cells. (**A**) A group of virus-like particles is visible inside a cytoplasmic vesicle (arrow) of an infected cell at 2 dpi. (**B**) Single particles were rarely found in the cell cytoplasm (arrow); extracellular virus-like particles are detected inside very small vesicles resembling exosomes (arrowheads). (**C**,**D**) Particles, likely viral particles, are also found in multivesicular bodies (MV) in infected cells at 10 dpi (arrows); lipid droplets (LD) are frequently observed. Nu = nucleus. Scale bars: (**A**,**D**) = 500 nm, (**B**) = 600 nm, (**C**) = 400 nm.

**Figure 3:**
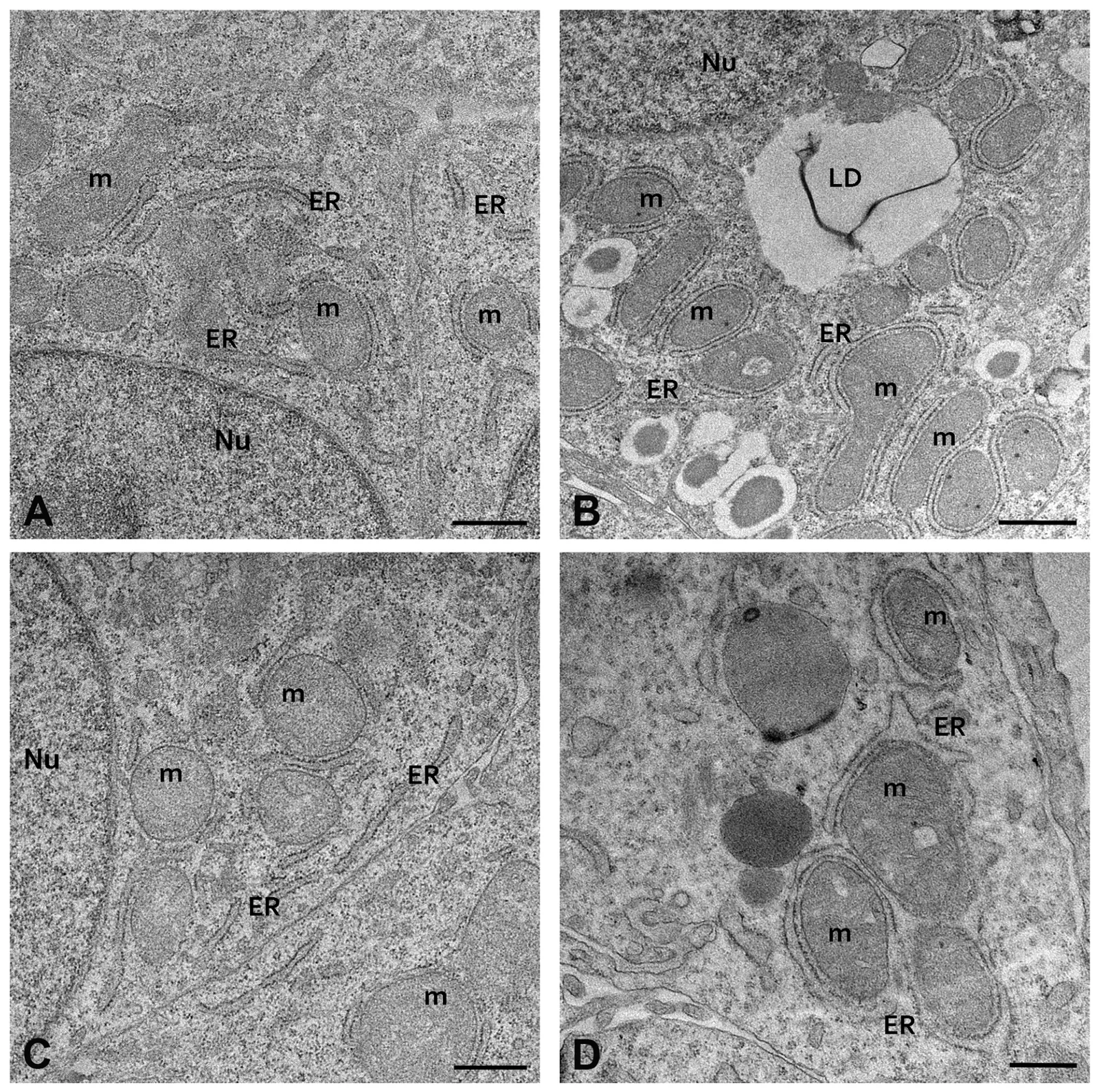
Representative electron microscopy images of mitochondrial morphology in uninfected and HBV-infected HepG2-NTCP cells. (**A**) Uninfected cells; (**B**) cells infected at 2 dpi; (**C**) cells infected at 5 dpi; and (**D**) cells infected at 10 dpi. Changes in mitochondrial size and in mitochondria–ER interactions are present during the temporal progression of HBV infection. Nu = nucleus, m = mitochondria, ER = Endoplasmic reticulum, LD = Lipid droplet. Scale Bars: (**A**) = 600 nm; (**B**) = 700 nm; (**C**,**D**) = 500 nm.

**Figure 4:**
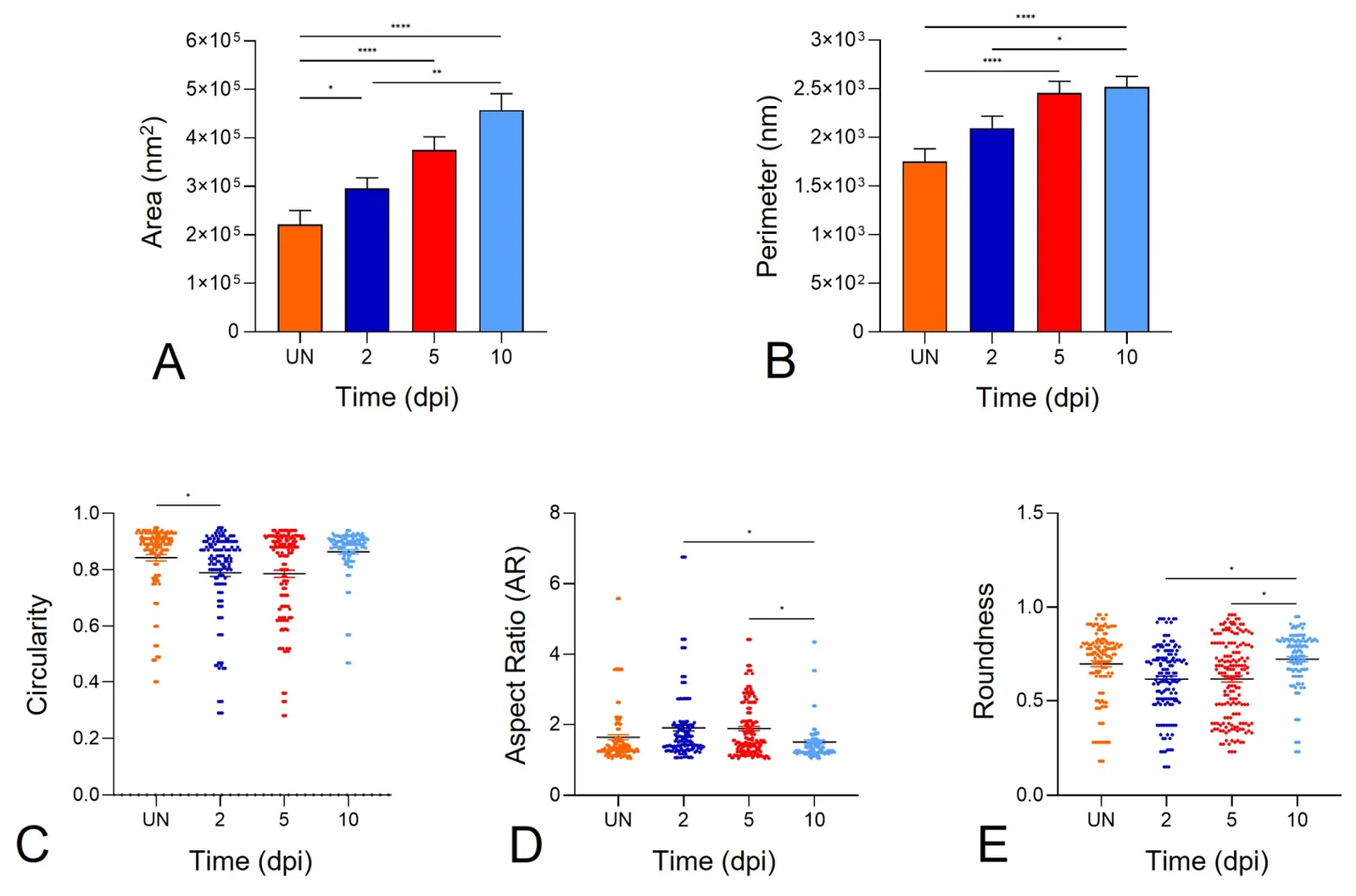
Electron microscopy morphometric analysis of mitochondria in uninfected (UN) and HBV-infected cells at different time points (2, 5, and 10 dpi). Morphological parameters were quantified by measuring area (**A**), perimeter (**B**), circularity (**C**), aspect ratio (AR) (**D**), and roundness (**E**). Data are presented as mean ± standard error of the mean (SEM). Statistical analyses were performed using the Kruskal–Wallis test. Image quantification was performed using ImageJ/Fiji software (fiji-windows-x64). Statistical significance is indicated as follows: *p* < 0.05 (*), *p* < 0.01 (**), and *p* < 0.0001 (****).

**Figure 5:**
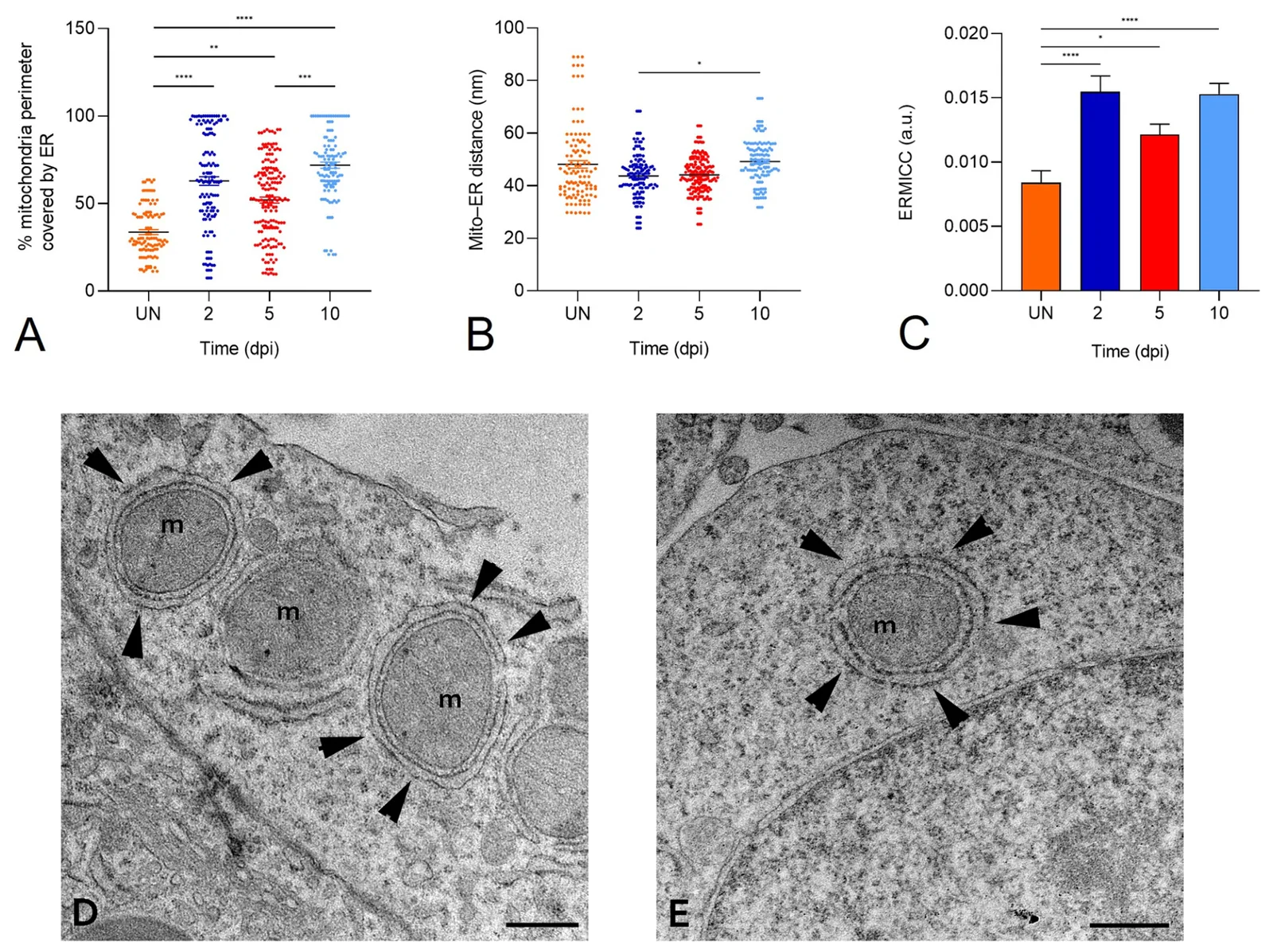
Ultrastructural morphometric analysis of mitochondria–ER interaction in uninfected (UN) and HBV-infected cells at different time points (2, 5, and 10 dpi). (**A**) Scatter plot showing the percentage of mitochondrial perimeter in contact with the ER. (**B**) Mitochondria–ER distance. (**C**) Column plot showing ERMICC index. Data are presented as mean ± SEM. Statistical analyses were performed using the Kruskal–Wallis test. Statistical significance is indicated as follows: *p* < 0.05 (*), *p* < 0.01 (**), *p* < 0.001 (***), and *p* < 0.0001 (****). (**D**,**E**) Representative electron micrographs showing mitochondria surrounded by ER membranes along the entire perimeter (arrowheads). Note: m = mitochondrion. Scale bar: (**D**) = 500 nm; (**E**) = 400 nm.

**Figure 6:**
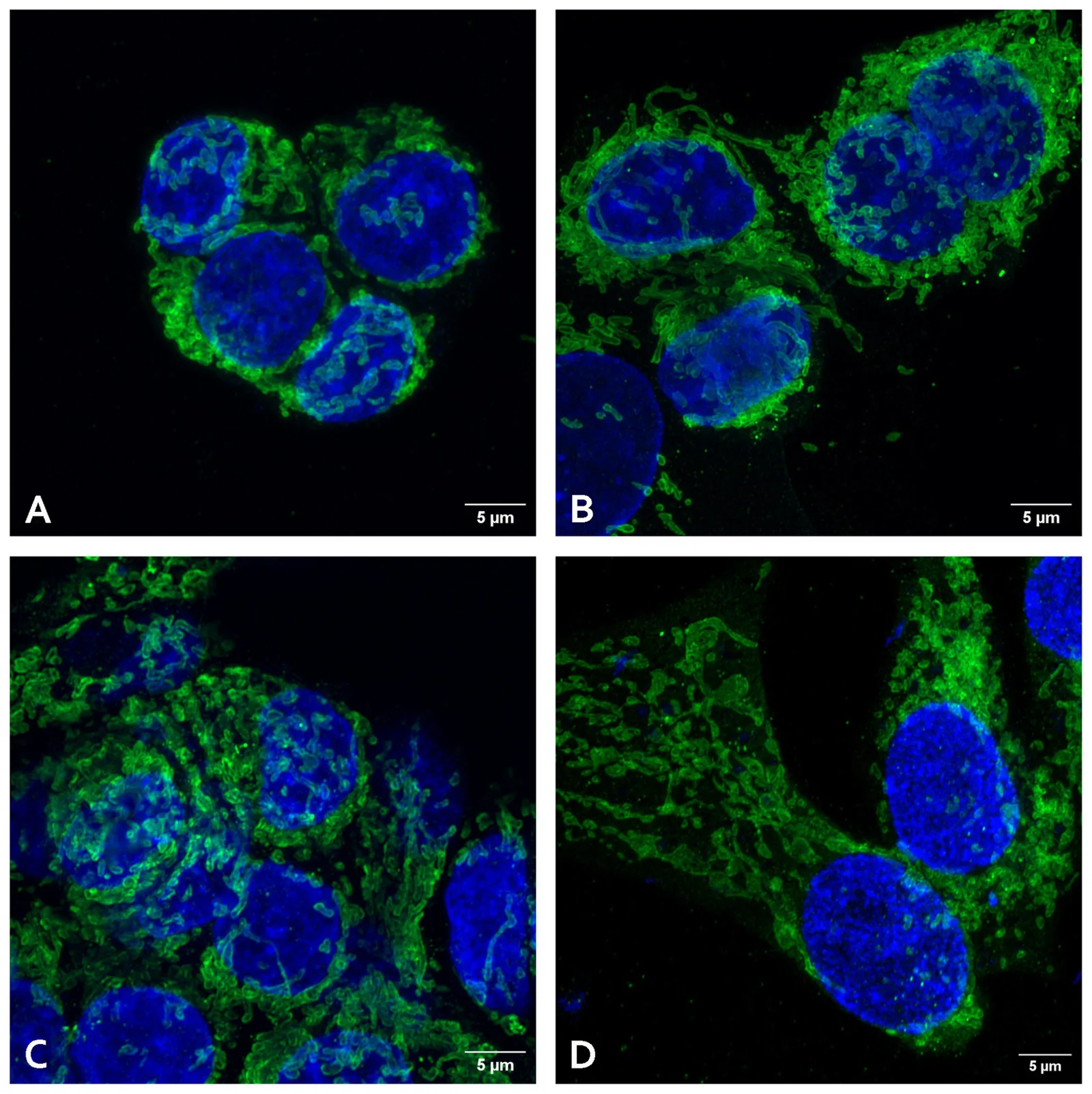
Confocal microscopy representative images of the mitochondrial network in uninfected and HBV-infected cells. (**A**) Uninfected cells; (**B**) HBV-infected cells at 2 dpi; (**C**) HBV-infected cells at 5 dpi; (**D**) HBV-infected cells at 10 dpi. Green staining shows localization of mitochondria. Changes in mitochondrial shape and in the organization of the mitochondrial network are visible, particularly at 10 dpi, as highlighted by more elongated mitochondrial shapes at 2 dpi and a more scattered distribution at 10 dpi. Staining: Nucleus Blue (DAPI); mitochondria Green (anti-Tom20/Alexa Fluor 488).

**Figure 7:**
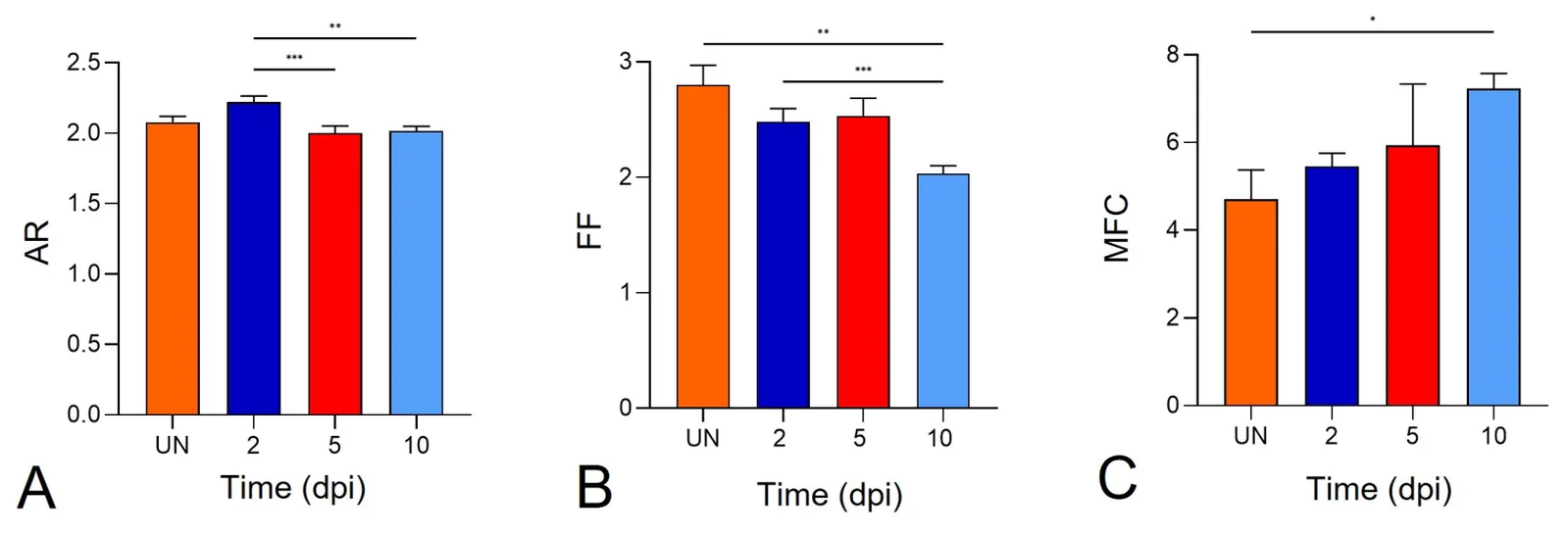
Confocal morphometric analysis of mitochondrial shape and network organization in uninfected (UN) and HBV-infected cells at different time points (2, 5, and 10 dpi). (**A**) Column plot showing quantification of: (**A**) Aspect Ratio (AR); (**B**) Form Factor (FF); (**C**) Mitochondrial Fragmentation Count (MFC), reflecting changes in the mitochondrial network. Data are presented as mean ± SEM. Statistical analyses were performed using the Kruskal–Wallis test. Image quantification was performed using ImageJ. Statistical significance is indicated as follows: *p* < 0.05 (*), *p* < 0.01 (**), *p* < 0.001 (***).

**Figure 8:**
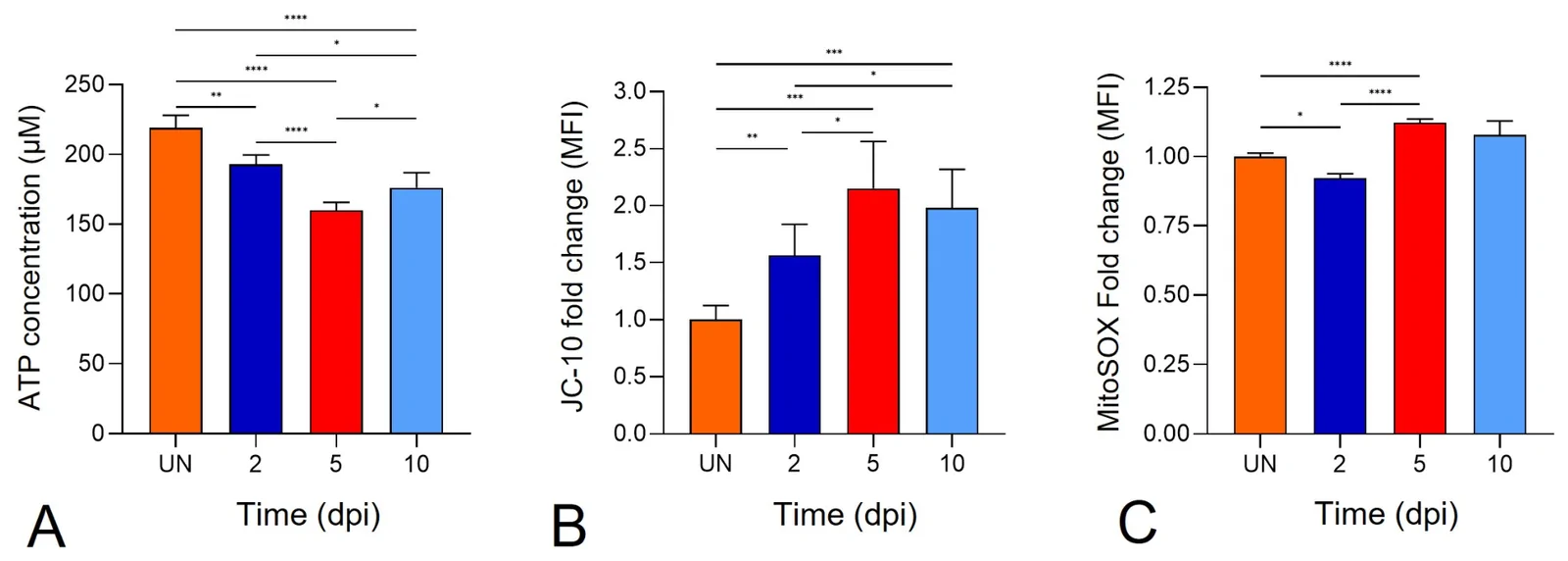
Mitochondrial functional analysis in uninfected (UN) and HBV-infected cells at different time points (2, 5, and 10 dpi). (**A**) Column plot illustrating ATP concentration. (**B**) Red/Green fluorescence (MFI), reflecting mitochondrial membrane potential. (**C**) Mitochondrial ROS (mtROS) production expressed as MFI fold change. Data are presented as mean ± SEM. Statistical analyses were performed using the Brown–Forsythe and Welch’s ANOVA test. Statistical significance is indicated as follows: *p* < 0.05 (*), *p* < 0.01 (**), *p* < 0.001 (***), and *p* < 0.0001 (****).

**Table 1:** Morphometric parameters of mitochondria measured in uninfected cells at 2, 5, and 10 dpi.

Morphometric Parameters	UN	2 dpi	5 dpi	10 dpi
Circularity	0.84 ± 0.019	0.79 ± 0.023 ^a^	0.78 ± 0.022	0.86 ± 0.015
Area (nm^2^)	221,327.78 ± 28,832.58	295,636.24 ± 22,167.41 ^b^	374,763.08 ± 27,445.71 ^d^	457,031.39 ± 34,184.39 ^c,e^
Perimeter (nm)	1753.85 ± 130.21	2095.67 ± 123.11	2475.00 ± 120.23 ^d^	2517.77 ± 109.30 ^e,f^

UN = uninfected, ^a^ *p* = 0.05 Uninfected vs. 2 dpi; ^b^ *p* < 0.05 Uninfected vs. 2 dpi; ^c^ *p* < 0.01 2 dpi vs. 10 dpi; ^d^ *p* < 0.0001 Uninfected vs. 5 dpi; ^e^ *p* < 0.0001 Uninfected vs. 10 dpi; ^f^ *p* < 0.05 2 dpi vs. 10 dpi.

**Table 2:** Mitochondria–ER association in uninfected cells and at 2, 5, and 10 dpi.

	UN	2 dpi	5 dpi	10 dpi
Associated	80.9%	93.6%	98.1% ^a^	100% ^b^
Not associated	19.1%	6.4%	1.9%	0%

UN = uninfected, ^a^ *p* < 0.01 Uninfected vs. 5 dpi; ^b^ *p* < 0.01 Uninfected vs. 10 dpi.

**Table 3:** Mitochondrial ER-envelopment in uninfected cells and at 2, 5, and 10 dpi.

	UN	2 dpi	5 dpi	10 dpi
Fully enveloped	0%	8.5%	0% ^b^	15% ^a,c^
Not fully enveloped	100%	91.5%	100%	85%

UN = uninfected, ^a^ *p* < 0.01 Uninfected vs. 10 dpi; ^b^ *p* < 0.05 2 dpi vs. 5 dpi; ^c^ *p* < 0.01 5 dpi vs. 10 dpi.

**Table 4:** Correlation of mitochondrial parameters with infection time.

		r	*p*-Value
Morphometric parameters	Mitochondrial area	0.44	<0.01
	Perimeter (nm)	0.40	<0.01
	% Apposition	0.60	<0.0001
	ERMICC ^a^	0.52	<0.001
	MFC ^b^	0.53	<0.01
Functional parameters	ATP	−0.71	<0.0001
	ΔΨm	0.74	<0.0001
	mtROS	0.45	<0.01

Data were analyzed using Spearman’s correlation. Note: r: Spearman’s correlation coefficient. *p* < 0.05 was set as the significance threshold. ^a^ ER–mitochondria Contact Coefficient; ^b^ Mitochondrial Fragmentation Count.

## Data Availability

The original contributions presented in the study are included in the article; further inquiries can be directed to the corresponding author.

## Supplementary Materials

The following supporting information can be downloaded at https://www.mdpi.com/article/10.3390/pathogens15101009/s1. Figure S1: Immunofluorescence assay of HBcAg (red) in uninfected (A) and HBV infected HepG2-NTCP cells at 2 dpi (B), 5 dpi (C) and 10 dpi (D).; Figure S2: Representative electron microscopy images of cell morphology of uninfected HepG2-NTCP cells; Video S1: 3D spatial mitochondrial distribution in uninfected cell; Video S2: 3D spatial mitochondrial distribution in nfected cells at 2 dpi; Video S3: 3D spatial mitochondrial distribution in infected cells at 5 dpi; Video S4: 3D spatial mitochondrial distribution in infected cells at 10 dpi.

## Abbreviations

The following abbreviations are used in this manuscript:

HBV	hepatitis B virus
ER	endoplasmic reticulum
mtROS	mitochondrial reactive oxygen species
(ΔΨm)	mitochondrial membrane potential
HCC	hepatocellular carcinoma
cccDNA	covalently closed circular DNA
CHB	chronic hepatitis B
TCA	tricarboxylic acid
OXPHOS	oxidative phosphorylation
ROS	reactive oxygen species
MERCs	mitochondria–ER contact sites
DMEM F-12	Dulbecco’s Modified Eagle’s medium/Nutrient Mixture F-12
ATCC	American Type Culture Collection
FBS	fetal bovine serum
NTCP	sodium taurocholate co-transporting polypeptide
DMEM	Dulbecco’s modified Eagle’s medium
VGE	viral genome equivalent
DMSO	dimethyl sulfoxide
PEG	polyethylene glycol
dpi	days post-infection
TEM	transmission electron microscopy
PBS	phosphate-buffered saline
BSA	bovine serum albumin
ROI	region of interest
AR	aspect ratio
ERMICC	ER–mitochondrial contact coefficient
3D	three-dimensional
FF	form factor
MFCs	mitochondrial fragmentation counts
MVBs	multivesicular bodies
